# Time for full inclusion of community actions in the response to AIDS

**DOI:** 10.7448/IAS.19.1.20712

**Published:** 2016-04-13

**Authors:** Chris J Collins, Matthew N Greenall, Chris Mallouris, Sally L Smith

**Affiliations:** 1 Community Mobilization Division, UNAIDS, Geneva, Switzerland; 2 Community, Rights and Gender Department, Global Fund to Fight AIDS, Tuberculosis, and Malaria, Geneva, Switzerland

**Keywords:** AIDS, HIV, community, services, health system, advocacy

## Abstract

**Introduction:**

Community action, including activism, advocacy and service delivery, has been crucially important in the global response to AIDS from the beginning of the epidemic and remains one of its defining features. This indispensable contribution has been increasingly acknowledged in strategic planning documents from UNAIDS, the Global Fund to fight AIDS, Tuberculosis and Malaria, the World Bank, the World Health Organization and other organizations. A growing body of literature demonstrates that community-based services can have measurable impact, serve populations that are not accessing public health services and reach people at scale.

**Discussion:**

Recognition of the powerful potential role of community has not translated into full incorporation of community responses in programme planning or financing, and communities are still not fully understood as true assets within overall systems for health. The diverse community contributions remain seriously underappreciated and under-resourced in national responses.

**Conclusions:**

It is time for a paradigm shift in how we think about, plan and finance community-based responses to HIV in order to achieve improved impact and move toward ending the epidemic. We must utilize the unique strengths of communities in creating resilient and sustainable systems for health. There are several priorities for immediate attention, including agreement on the need to nurture truly comprehensive systems for health that include public, private and community activities; re-examination of donor and national funding processes to ensure community is strategically included; improvement of data systems to capture the full spectrum of health services; and improved accountability frameworks for overall health systems. Health planning and financing approaches run by governments and donors should institutionalize consideration of how public, community and private health services can strategically contribute to meeting service needs and accomplishing public health targets.

## Introduction

Community action, including activism, advocacy and service delivery, has been crucially important in the global response to AIDS from the beginning of the epidemic and remains one of its defining features. This indispensable contribution has been increasingly acknowledged. The Strategic Investment Framework identifies community mobilization and community-led services as “critical enablers” of effective responses [1]; UNAIDS Fast Track targets call for increasing reliance on community-provided testing and treatment [2]; and the Global Fund to Fight AIDS, Tuberculosis and Malaria encourages incorporation of community-led services and advocacy into grants. World Health Organization guidelines strongly recommend utilization of community-based approaches to HIV testing, counselling and delivery of antiretroviral therapy [3–5].

Unfortunately, these policy commitments have not translated into full incorporation of community responses in programme planning or financing, and communities are too often not recognized as true assets within overall systems for health. It is time for a paradigm shift in how we think about, plan and finance community-based responses to HIV in order to achieve improved impact and move toward ending the epidemic. We must utilize the unique strengths of communities in creating resilient and sustainable systems for health to accomplish HIV and other public health goals.

## Discussion

*Community-based initiatives* refer to activities in which community members are actively engaged, ranging from involvement in governance and design of programmes to delivery of services. Community-based service delivery can allow services to be more responsive to health consumer needs, adapt programmes to specific social contexts and build trust with communities. Community-based initiatives can fill gaps in, and create valuable support for, formal public health systems, either in close coordination with public health systems or through stand-alone services. Community-led and community-centred services can be highly effective at reaching groups that are typically excluded (such as criminalized and stigmatized groups), monitoring how programmes are delivered and providing rapid feedback, and mobilizing communities to identify problems and solutions in accessing services.

For efficacious interventions to be effective in the field, people need a safe, supportive environment to access services, and in many cases other needs must be addressed beyond simple delivery of a medication or other intervention. Meaningful engagement of communities is necessary to ensure clinical support is provided in a holistic environment that can address these needs. Specific good practice examples of community initiatives on advocacy, services, research and financing have been documented [6].

A growing body of literature demonstrates that community-based services have measurable impact, serve populations that are not accessing public health services [7] and can reach people at scale [8,9]. A 2012 World Bank study identified community-based efforts as a “cornerstone” of the response to AIDS [10]. Médecins Sans Frontières has documented how community-based HIV treatment delivery increases retention in care and lowers costs [11]. By reducing the burden faced by healthcare facilities, community-led services can help to support overburdened health systems. The recent *Lancet* series on faith and healthcare documents the important role of faith-based providers in contributing to service delivery through their facilities and widespread community initiatives, particularly where health systems are fragile or weakened [12].

Activism, advocacy and accountability work are also essential contributions of community action, helping create the enabling environment for a more robust response. Communities play critical roles in generating demand for quality services, advancing the needs of marginalized groups and bringing to light emerging needs and priorities. Prominent leaders in the AIDS response have argued that “activism constitutes a global public good, deserving investment commensurate with the role it plays in improving health outcomes” [13].

Experience from both HIV and the Ebola crisis has made clear that community engagement in the design and delivery of health services is crucial, given the complex interrelationships between culture, traditional healthcare practices, and stigma and discrimination, all of which affect vulnerability to infection and uptake of health services [14]. In the case of Ebola, resistance occurred when traditional public health approaches were used to attempt to change long-standing burial practices that spread the disease. When community and religious leaders were engaged in discussions to rewrite the safe and dignified burial protocol (incorporating elements of traditional and religious practices that were culturally important to families, yet safe) and included in the burial teams, then practices changed. This demonstrates a clear pathway between community engagement and change in health outcomes.

These diverse community contributions remain seriously underappreciated and under-resourced in national responses. For example, the Global Fund's Technical Review Panel has raised concern about the lack of community systems interventions in the majority of the concept notes reviewed in a recent grant round [15]. The great majority of financing for community-based AIDS services in lower and middle-income countries still comes from external funders, in particular for key populations, and an increasing share of national responses funded by domestic resources in middle-income countries risks further decreasing investments in these areas. The added value of community action is also less clear to national-level decision makers in an era when the response to AIDS increasingly emphasizes biomedical tools. As a result, community action at scale is seldom incorporated into national health systems planning and financing.

## Conclusions

A more systematic approach to incorporating community actions is particularly critical now given the challenges ahead in the AIDS response. These challenges include an urgent need to do much better in reaching groups that are socially and legally marginalized, providing support to help retain people in treatment, rapidly scaling up HIV testing and linkage to care, and more effectively tackling stigma and discrimination – all areas where communities have particular strengths.

There are several priorities for immediate attention. First, broad agreement is needed on promoting truly comprehensive systems for health that incorporate public, community and private services. Separating community from the system for health leads to disjointed responses that are centred on sectors, not people. This means that analysis of a health system's strengths and weaknesses must review the complex ecosystem of actors that have a role in health rather than focusing only on formalized systems, facilities and healthcare workers. Health data should no longer focus only on the activities and roles of the formal or biomedical sector.

Second, global funders and country governments need to re-examine their processes for planning and financing comprehensive HIV services to ensure that the strategic roles of communities are adequately resourced. Health planning and financing approaches run by governments and donors should institutionalize consideration of how public, community and private health services can strategically contribute to meeting service needs and accomplishing public health targets. Building on the expanded analysis described above, plans for strengthening systems for health should aim for investments in the whole range of actors and assets that are critical to improving health. A person- or community-centred approach will aim to create and strengthen systems that respond to people's needs, rather than a sector's needs.

Third, data systems must be improved to reflect the diversity of services and providers within a system for health. Currently, health data primarily capture information on facility-based and public health services. Fourth, improved accountability frameworks for overall health systems are important to advancing the reach, equity and quality of services. For example, the Stop Stock Outs programme spearheaded by South Africa's Treatment Action Campaign and other organizations engages community members in monitoring and reporting essential medicine stock-outs in the country. Community-led advocacy and accountability mechanisms need systematic support to enable citizens to monitor satisfaction and access to services, to identify problems and propose solutions.

Accessible, equitable and sustainable health services are essential to achieving the ultimate goal of dramatically reduced HIV-related incidence, morbidity and mortality and the other health benefits that will accompany advances against the AIDS epidemic. Systematic inclusion of community responses in health planning and financing is required to achieve that goal, and these responses should be financed and supported consistent with their essential role in ending AIDS and advancing health for all.
